# Biochemical Characterization and Molecular Determination of Estrogen Receptor-α (ESR1 PvuII-rs2234693 T>C) and MiRNA-146a (rs2910164 C>G) Polymorphic Gene Variations and Their Association with the Risk of Polycystic Ovary Syndrome

**DOI:** 10.3390/ijerph19053114

**Published:** 2022-03-06

**Authors:** Rashid Mir, Faris J. Tayeb, Jameel Barnawi, Mohammed M. Jalal, Nizar H. Saeedi, Abdullah Hamadi, Malik A. Altayar, Sanad E. Alshammari, Nabil Mtiraoui, Mohammed Eltigani Ali, Faisel M. Abu Duhier, Mohammad Fahad Ullah

**Affiliations:** 1 Faculty of Applied Medical Science, University of Tabuk, Tabuk 71491, Saudi Arabia; jbarnawi@ut.edu.sa (J.B.); fabu-duhier@ut.edu.sa (F.M.A.D.); 2 Department of Medical Laboratory Technology, Faculty of Applied Medical Science, University of Tabuk, Tabuk 71491, Saudi Arabia; f.tayeb@ut.edu.sa (F.J.T.); mjalal@ut.edu.sa (M.M.J.); nsaeedi@ut.edu.sa (N.H.S.); a.aldhafri@ut.edu.sa (A.H.); maltayar@ut.edu.sa (M.A.A.); 3 Department of Pharmacology & Toxicology, Faculty of Pharmacy, University of Hail, Hail 55476, Saudi Arabia; se.alshammari@uoh.edu.sa; 4 Laboratory of Human Genome and Multifactorial Diseases, Faculty of Pharmacy, University of Monastir, Monastir 5000, Tunisia; mtiraouinabil@yahoo.fr; 5 King Salman Military Hospital, Tabuk 47512, Saudi Arabia; tigoalgarbawi@yahoo.com

**Keywords:** polycystic ovary syndrome, gene variations, polymorphism, endocrine

## Abstract

Polycystic ovary syndrome (PCOS) is regarded as one of the most frequently encountered endocrine disorders and affects millions of young women worldwide, resulting in an array of complex metabolic alterations and reproductive failure. PCOS is a risk factor for diabetes mellitus, obstructive sleep apnea, obesity and depression in patients. Estrogen receptors (ESRs) are significant candidates in endocrine function and ovarian response in women. Moreover, microRNAs and long non-coding RNAs are emerging as principal mediators of gene expression and epigenetic pathways in various disease states. This study has characterized the clinical parameters in PCOS patients with comprehensive biochemical profiling compared to healthy controls and further examined the influence of allelic variations for estrogen receptor-α (ESR1 PvuII-rs2234693 T>C) and miRNA-146a (rs2910164 C>G) gene polymorphism on the risk of and susceptibility to PCOS. In this case-control study, we have used amplification refractory mutation specific (ARMS)-PCR to detect and determine the presence of these polymorphic variants in the study subjects. Our results demonstrated that most of the biochemical markers, which were analyzed in the study, show statistically significant alterations in PCOS patients, including fasting glucose, free insulin, HOMA-IR, LDL, HDL, cholesterol and hormones such as FSH, LH, testosterone and progesterone, which correlate with the established biochemical alterations in the disorder. Further, it is reported that for estrogen receptor-α (ESR1 PvuII-rs2234693 T>C), the frequency of the T allele (fT) was significantly higher among patients (0.64 vs. 0.44) compared to controls, while the frequency of the C allele (fC) was lower in patients (0.36 vs. 0.56) compared to controls. However, it was found that there was no association of an increased risk of PCOS with the ESR1 PvuII-rs2234693 C>T gene polymorphism. On the contrary, the study found strong association of miRNA-146a (rs2910164 C>G) gene polymorphism with an enhanced risk of PCOS. The frequency of the C allele (fC) was significantly higher among patients (0.52 vs. 0.36) compared to controls. The frequency of the G allele (fG) was found to be lower in patients (0.48 vs. 0.64) compared to controls. The codominant, dominant and recessive models display a statistically significant association of polymorphic variations with PCOS. Moreover, the G allele was associated strongly with PCOS susceptibility with an OR = 1.92 (95%) CI = (1.300–2.859), RR = 1.38 (1.130–1.691) *p*-value < 0.001.

## 1. Introduction

Polycystic ovary syndrome (PCOS) is one of the most frequently encountered endocrine disorders and affects millions of young women worldwide, resulting in an array of complex metabolic alterations and reproductive failure [1,2,3]. Various symptoms, including irregular menses and hirsutism characterize it, and it can lead to infertility. Women diagnosed with PCOS are more likely to develop diabetes mellitus, obstructive sleep apnea, obesity and depression, which worsens with the severity of the disease and advanced age [4]. 

ESRs (estrogen receptors) are significant players in the ovarian response to follicle-stimulating hormone (FSH) because direct effects of estrogens on the growth of the follicle, its maturation, and subsequent oocyte release are well established [5]. In addition to folliculogenesis, estrogens are essential in endometrial preparation for implantation [6]. Estrogen receptors are transcription factor activators, consisting of numerous domains that are necessary for hormone binding, DNA binding, transcription activation and in mediating the estrogen transduction pathway [7]. Humans have two estrogen receptors, ERα (6q25)/ERβ (14q22), which are encoded by the ESR1/ESR2 genes, respectively. Estrogens’ proliferative activities in folliculogenesis are mediated by ERα (dominant expression in the theca layer). However, the differentiation and anti-proliferative functions are executed via granulosa ERβ which are essential for the development of maturing follicles to reach the antral stage [8,9]. Genetic variations in critical gene loci contribute to the variations in the risk of disease and its severity, effectiveness of the treatment and the expected prognosis. For instance, several studies have shown that genetic variability in ESR genes influences the effectiveness of controlled ovarian stimulation (COS) [10,11,12,13,14,15]. Interestingly, the first pharmacogenetic technique employed in COH/IVF in 1997 was based on polymorphisms in the ESR1 gene [10]. The ESR1 gene is pleiotropic, with over 2200 SNPs discovered so far, whereas approximately 720 SNPs have been known to be associated with ESR2 gene. Furthermore, a link between the G allele and greater estradiol levels have been reported and such ESR1 gene variants are emerging as predictors of ovarian stimulation outcomes in IVF treatments [13]. Studies on granulosa cells and theca cells obtained from the ovaries of PCOS patients show significant alterations in the expression of ERα and ERβ compared to control ovaries, which is considered an important factor in retarded follicular development and ovulatory failure [16,17]. Theca cells, which predominantly express ERα contribute to the fulfilment of the production of androgens required by the developing follicle that is converted into estrogens by the granulosa cells, and thus have a fundamental role in androgen excess observed in the pathophysiology of PCOS [18]. 

In PCOS, epigenetic modifications can contribute to transgenerational inheritance [19]. For instance, diabetes and obesity can result after exposing three generations to an unfavorable metabolic environment. It may also chronically affect women’s reproductive outcome [20]. MicroRNAs and long non-coding RNAs are emerging as principal mediators of gene expression and epigenetic pathways [21,22]. Consequently, miRNAs are linked to various pathological conditions such as insulin insensitivity, inflammation and metabolic disorders. Likewise, some correspond to syndrome-related illnesses such as diabetes, cancer and PCOS [23]. Evidence in the literature demonstrates the warped-up expression of certain miRNAs in ovarian granulosa and theca cells; which are thought to play a vital role in the development of PCOS [24]. In a recent meta-analysis many ncRNAs with altered levels were reported in serum, plasma, granulosa cells and follicular fluid from PCOS patients; in particular miR- 93 expression was upregulated in PCOS patients, without heterogeneity among remaining studies [25]. Several miRNAs are known to play key roles in the pathogenesis of PCOS; one of these is miR- 222, which has been linked to insulin resistance and metabolic syndrome and it is also known for its high-level correlation to gestational diabetes [23]. Another microRNA of significance in PCOS is miR- 146a, which is a potent regulator of the degeneration and atresia of human ovarian follicles. It can regulate the apoptosis of human granulosa cells (GCs) by directly activating TNF-associated factor 6 (TRAF6) and interleukin-1 receptor-associated kinase 1 (IRAK1) [23]. Serum levels of miR- 146a have been shown to be elevated in PCOS patients and its association with altered testosterone levels has also been established [26]. Recent studies have shown that endometrial receptivity and placentation requires certain modulation of immune response and microRNAs such as miR-146a have a regulatory role in key gene networks operating in the mammalian reproductive pathway for conception [27]. Several microRNAs were detected to be differentially expressed between PCOS women (anovulatory) and control women (in follicular phase) matched for BMI, and these were found to be associated with pathways related to reproductive dysfunction [28]. 

Thus, the presence of a multitude of metabolic impairments in PCOS and the resulting metabolic and reproductive failures, and treatment outcomes might be attributed to an array of warped-up regulatory mechanisms. It is believed that these regulatory mechanisms are subject to cues from environmental and genetic factors including the epigenetic blueprint and gene polymorphisms, which might be related to the risk of various diseases. Recent observations by the experts have noted that currently the guidelines on the assessment and management strategies of PCOS are still vague due to low to moderate quality evidence [29]. Thus, considerable refinement is required in various aspects of disease diagnosis and management including diagnostic criteria for accuracy of diagnosis, lifestyle modification and evidence based therapy. Additionally, a consensus resolution also recognized PCOS as a major health concern that effects general health, sexual function and quality of life in women, and recommended research initiatives to explore novel aspects and benefits for the management of the disease [30]. The important roles of genetic and epigenetic factors have also been suggested as mediators of the disease since it has been observed that the symptomatic indications of PCOS appear in early life in female infants of PCOS carriers [30]. 

The current study focuses on the pathogenesis of PCOS and quantification of the risk of and susceptibility to disease in relation to ESR1 PvuII-rs2234693 T>C and microRNA-164a-rs2910164 C>G gene polymorphisms. In gene variation studies, the two most common polymorphisms in ESR1 are located in the first intron of the ESR1 gene; 397 and 351 base pairs upstream of exon 2, which are identified by PvuII and XbaI restriction endonucleases, respectively [31]. The polymorphism rs2234693 (397T>C) relates to the PvuII restriction site, whereas rs9340799 (351A>G) relates to the XbaI restriction site. It is a SNP upstream of the estrogen alpha receptor ESR1 gene, and is sometimes referred to as the −397 T>C variation. The ESR1 PvuII (rs2234693 T>C) polymorphism can affect the ESR1 transcription, thus could be involved with the disease risk and susceptibility. Furthermore, the rs2910164 polymorphism is a functional variant of miRNA-146a, resulting from a nucleotide substitution from G to C [32]. The enhanced expression of miRNA-146a caused by the rs2910164 polymorphism has been associated with SORT1 dependent alterations in lipid metabolism [33,34].

## 2. Material and Method

### 2.1. Study Subjects

The guidelines of the 2003 Rotterdam Criteria were used in the study to confirm the clinical cases of PCOS [35]. In order to have an ethnically conserved genetic variation study only Arabs (Saudis) were included in the study while non-Saudi Arabs, non-Arabs or recently naturalized Arabs were excluded as subjects. This is part of a large PCOS based genome-wide study in which 217 subjects that included 102 PCOS patients and 115 gender-matched control were enrolled at King Salman Military Hospital, Tabuk (KSA).

### 2.2. Biochemical Characterization

The patients and control subjects underwent biochemical profiling which included a hormonal profile, lipid profile and markers for type 2 diabetes such as free insulin, fasting glucose and HOMA-IR, which have been conventionally altered in PCOS patients. Serum levels of different hormones including progesterone, TSH, FSH, LH, estradiol and testosterone, were determined with their respective ELISA kits [36]. A lipid profile for LDL, HDL, TAGs and cholesterol was generated by colorimetric estimations (Cobas Integra 800; Roche, Germany). A hexokinase kit (Cobas Integra 800; Roche, Germany) was used to measure fasting glucose. An ELISA-DRG EIA kit was used to determine total insulin as per the vendor’s instructions. A HOMA calculator (www.dtu.ox.ac.uk/homa/index, accessed on 15 January 2022) determined the HOMA-IR index.

### 2.3. Genomic DNA Extraction 

A 3 ml sample of peripheral blood was collected by venipuncture in the EDTA tubes of each study subject in both the patient and control groups. The extraction of genomic DNA was performed with a DNeasy Blood Kit (Qiagen, Hilden, Germany) as per the vendor’s specifications. The extracted DNA was dissolved in 100 µL of TE buffer. The quality of DNA was checked on 1% gel electrophoresis. The DNA was quantified by NanoDrop™ (Thermo Scientific, Waltham, MA, USA). The DNA samples also had a qualitative check for the purity by determining optical density (OD) at 260 and 280. The ratios A_260/_A_280_ that ranged from 1.83–1.99 indicated good quality DNA.

### 2.4. Genotyping of Estrogen Receptor 1-(ESR1 PvuII-rs2234693 T>C) and miR- 146a-(rs2910164 C>G)

Both ESR1 PvuII-rs2234693 T>C and miR- 146a-rs2910164 C>G genotyping was done by optimizing the amplification–refractory mutation system (ARMS). The primers to assess ESR1 PvuII (rs2234693 T>C) and miR- 146a-rs2910164 C>G polymorphisms [37]. (Table 1) were designed using Primer3 Input (version 0.4.0), Whitehead Institute for Biomedical Research, Steve Rozen, Maido Remm, Triinu Koressaar and Helen Skaletsky. A gradient PCR was performed in a reaction volume of 25 uL containing template DNA (50 ng), Fo-0.25 µL, Ro-0.25 µL of 25 pmol of each primers, FI−0.25 uL, RI−0.25 uL of 25 pmol of each primers and 10 µL from GoTaq^®^ Green Master Mix (cat no M7122) (Promega, Madison, WI, USA). Gradient PCR is a method which can be employed to obtain an optimal annealing temperature in a single experimental setup, avoiding several steps. The optimum temperature was found to be 60 °C for ESR1 PvuII (rs2234693 T>C) and 62 °C for miR- 146a-rs2910164 C>G in the range of 55 °C to 64 °C obtained in a gradient PCR thermocycler. The number of cycles was raised from 35 to 45, which enhanced the yields of all three PCR products. 

*ESR1 PvuII (rs2234693 T>C):* The cycling conditions included a hot start at 95 °C for 8 min, 40 amplification cycles at 95 °C/35 s, 60 °C/35 s and 72 °C/45 s with one elongation step at 72 °C/ 10 min and storage at 4 °C. *miR- 146a-rs2910164 C>G:* The cycling conditions included a hot start at 95 °C for 8 min, 40 amplification cycles at 95 °C/35 s, 62 °C/35 s, and 72 °C/45 s with one elongation step at 72 °C/10 min and storage at 4 °C.

### 2.5. Gel Electrophoresis and PCR Product Visualization

The amplified PCR products were resolved by agarose gel electrophoresis (2%), with 0.5 μg/mL EtBr and visualized using a UV transilluminator.

**Figure 1:**
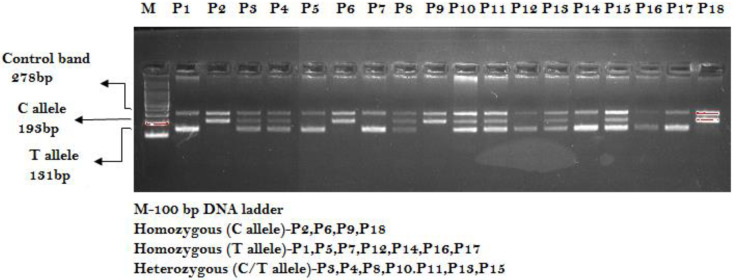
Genotyping of estrogen receptor-1 (ESR1 PvuII-rs2234693 T>C) by ARMS-PCR in PCOS patients.

**Figure 2:**
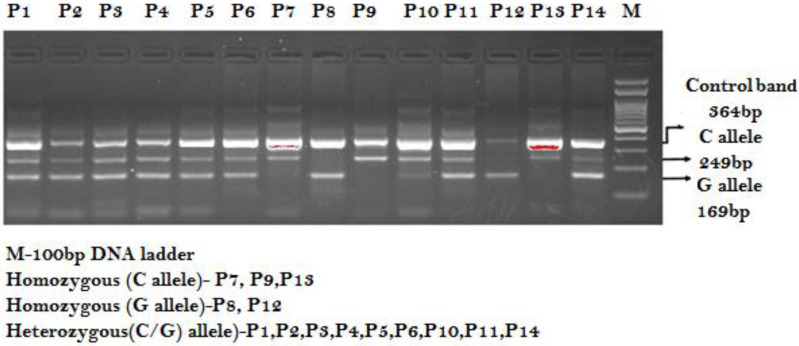
Genotyping of miR- 146a rs2910164 C>G by ARMS-PCR in PCOS patients.

1. **ESR1 PvuII-rs2234693 T>C amplification:** Primers Fo/Ro flank the intron of the ESR1 PvuII-rs2234693 T>C amplifying into a band of 278 bp as a qualitative and quantitative DNA experimental control. Primers Fwt/Ro provide the amplification of the T allele (wild-type allele), with a band of 131 bp, and primers Fo/Rmt amplify into a band of 193 bp that represents the mutant allele (C allele) as shown in Figure 1.
2. **MicroRNA-146a rs2910164 C>G amplification**: Primers Fo/Ro flank the exon of the miR- 146a-rs2910164 C>G gene, amplifying into a band of 364 bp as a qualitative and quantitative DNA experimental control. Primers FI/Ro provide the amplification of the C allele (wild-type allele) with a band of 169 bp, and primers Fo/RI amplify into a band of 249 bp that represents the mutant allele (G allele) as shown in Figure 2.

### 2.6. Statistical Analysis

Statistical analysis was performed using the SPSS 16.0 software (Chicago, IL, USA). *Hardy-Weinberg disequilibrium (HWD):* Deviation from Hardy–Weinberg disequilibrium (HWD) was determined by a Chi-square (χ^2^) goodness-of-fit test. The comparison of group differences were demonstrated using Student’s two-sample t-test or ANOVA for continuous variables and a Chi-square test for categorical variables. We found that the ESR1 PvuII-rs2234693 T>C and miR- 146a rs2910164 C>G frequency were in compliance with the HWE, considering all the participants. No deviation was observed in the HWE in the patient group (all *p* > 0.05) and similarly no deviation was observed in the HWE in the controls (all *p* > 0.05), considering the genotype distributions and allele frequencies. *Chi-square analysis and Fisher exact test:* Chi-square and Fisher exact tests were performed to compare miR- 146a rs2910164 C>G and ESR1 PvuII-rs2234693 T>C genotyping frequency with several biochemical parameters. Multivariate analysis: The associations between the genotypes and the risk of PCOS for patients was estimated through unconditional logistic regression. Adjusted odds ratios (OR) and 95% confidence intervals (95% CI) associated with the risk of PCOS, were analyzed by logistic regression after controlling for a number of covariates and comparison with the reference group (healthy controls). Multivariate analysis was used to study the link between miR- 146a rs2910164 C>G and ESR1 PvuII-rs2234693 T>C genotyping and the susceptibility to disease in terms of odds ratios (ORs), risk ratios (RRs) and risk differences (RDs) with 95% confidence intervals (CIs) [38,39].

## 3. Results

### 3.1. Clinically Altered Profile of Biochemical Markers in PCOS Patients

Most of the biochemical markers, which were analyzed in the study, show statistically significant alterations in PCOS patients. The mean age of patients and controls at the time of inclusion in the study was approximately 27 years and there was no significant variation in both groups. As displayed in Table 2, the fasting glucose, free insulin and HOMA-IR values were significantly elevated in patients, and results confirmed the concurrent diagnosis of the PCOS patients with type 2 diabetes. The lipid profile for serum cholesterol, TAGs, LDL and HDL showed higher levels in patients with significant differences from the control group. Significant alterations were observed in the levels of progesterone, follicle stimulating hormone and luteinizing hormone in the patient group. The level of testosterone was also higher in PCOS patients which demonstrated hyperandrogenism, which is a prominent feature of this endocrine and metabolic syndrome. Differences in the mean body mass index were also significant in patients and were related to the altered lipid profiles. 

### 3.2. Statistical Comparisons between Patients and Controls (p-Values) for ESR1 PvuII-rs2234693 T>C Genotypes

Our results show that the ESR1 PvuII-rs2234693 T>C frequency among all the participating subjects is in compliance with the HWE. The genotype distributions and allele frequencies of the SNPs located in the ESR1 PvuII-rs2234693 T>C showed no deviation in HWE in the PCOS patient group (all *p* > 0.05) (χ^2^ = 2.24, *p* ≤ 0.13) or in healthy controls (all *p* > 0.05) (χ^2^ = 0.52, *p* ≤ 0.47). Thus, 10% samples from normal control group were randomly chosen to assess the genotyping results, showing that the accuracy rate was more than 99%.

### 3.3. Allele and Genotype Frequency of ESR1 PvuII-rs2234693 C>T Gene Polymorphism in Cases and Controls

In PCOS patients, the CC, CT and TT genotype frequencies were 9.80%, 50.98% and 39.21%, respectively. The genotype frequencies in healthy controls for CC, CT and TT were 11.30%, 56.52%, and 32.17%, respectively (Table 3). It was noted that the distribution of ESR1 PvuII-rs2234693 C>T genotypes in patients and controls was not significantly different (*p* = 0.55). However, the frequency of the T allele (fT) was significantly greater among patients (0.64 vs. 0.44) compared to controls, whereas the frequency of the C allele (fC) was lower in patients (0.36 vs. 0.56) compared to the controls, as exhibited in Table 3.

### 3.4. Multivariate Analysis of ESR1 PvuII-rs2234693 C>T Gene Polymorphism between PCOS Patients and Healthy Controls

As reported in Table 4, there was no association of an increased risk of PCOS with the ESR1 PvuII-rs2234693 C>T gene polymorphism.

Our results demonstrated that in the codominant model, the ER-CT and TT genotypes of the estrogen receptor-1 (ESR1 PvuII-rs2234693 T>C) gene have no association with the risk and susceptibility to PCOS with OR = 1.04 (95%) CI = (0.422 to 2.56), RR = 1.01 (0.686 to 1.507), *p* < 0.93. In the dominant inheritance model, ER-CC vs. ER-(CT + TT) genotype was not associated with susceptibility to the disease with OR = 1.17 (95%) CI = (0.49 to 2.802), RR = 1.07 (0.733 to 1.576), *p* < 0.071. Furthermore, no significance was observed in the recessive inheritance model ER-TT vs. (CC + CT) with regard to the association to PCOS disease with OR = 1.36 (95%) CI = (0.77–2.37), RR = 1.55 (0.88–1.52), *p* < 0.27. In terms of allelic comparisons, the CC allele of the estrogen receptor gene polymorphism was not associated with susceptibility to PCOS with OR = 1.36 (95%) CI = (0.77 to 2.375), RR = 1.01 (0.909 to 1.302), *p* < 0.35.

### 3.5. Statistical Comparisons between Patients and Controls (p Values) for miR- 146a rs2910164 C>G Genotypes

Our results show that miR- 146a rs2910164 C>G frequency among all the participating subjects is in compliance with the HWE. There was no deviation observed in HWE for the genotype distributions and allele frequencies of the SNPs located in the miR- 146a rs2910164 C>G in the PCOS patient group (all *p* > 0.05) (χ^2^ = 2.34 *p* ≤ 0.13) and in the controls (all *p* > 0.05) (χ^2^ = 0.55 *p* ≤ 0.48). Thus, 10% samples from the normal control group were randomly chosen to assess the genotyping results, demonstrating that the accuracy rate was more than 99%.

### 3.6. Allele and Genotype Frequency of Hsa-miR- 146a rs2910164 C>G Gene Polymorphism in Cases and Controls

As reported in Table 5, in PCOS patients, the CC, CG and GG genotype frequencies were 30%, 43% and 27%, respectively. In healthy controls the frequencies of CC, CG and GG genotype were 16.82%, 37.38%, and 45.79%, respectively. It was shown that the distribution of miR- 146a rs2910164C>G genotypes in patients and controls was significantly different (*p* = 0.024). Further, the frequency of the C allele (fC) was significantly greater in patients when compared to the controls (0.52 vs. 0.36). However, the frequency of the G allele (fG) was lower in patients than in controls (0.48 vs. 0.64).

### 3.7. Multivariate Analysis to Determine the Association between miR- 146a rs2910164 C>G Genotypes and Risk to PCOS

A multivariate analysis based on logistic regression was carried out for each group to determine the association between hsa-miR- 146a rs2910164 C>G genotypes and risk to PCOS. The data displayed in Table 6 showed that in the codominant model, there was a strong association of miR- 146a GG genotype with an increased PCOS susceptibility with OR = 3.02 (95%) CI = (1.429–6.401), RR = 1.71 (1.150–2.568), *p* < 0.003. 

Moreover, it was observed that there was a strong association between the miR- 146-CC genotype vs. the miR- 146-(CG + GG) genotype in the dominant inheritance model which might lead to an increased PCOS susceptibility with OR = 2.28 (95%), CI = (1.275–4.090), RR = 1.45 (1.129–1.878), *p* < 0.003. Additionally, there was a strong association observed between the miR- 146-(CC + GC) genotype vs. the miR- 146-CG genotype in the recessive inheritance model predisposing the individuals to an increased risk of PCOS with OR = 2.11(95%), CI = (1.092–4.112), RR = 1.49 (1.010−2.205) and *p* < 0.026. In terms of allelic comparison, it was the G allele that was observed to be strongly associated with PCOS susceptibility with an OR = 1.92 (95%), CI = (1.300–2.859), RR = 1.38 (1.130–1.691) and *p*-value < 0.001.

## 4. Discussion

The symptomatic and clinical features of PCOS patients show a marked variation in a number of parameters which makes it a complex disease due to heterogeneity and aberrations in multiple metabolic and molecular pathways which are influenced by multigenic, epigenetic, endocrine and environmental factors [40]. The disease is manifested by the presence of hyperglycemia, insulin resistance, obesity and altered endocrinology in a large proportion of patients. In our study, the subjects, which included both patients and controls, were of the same age group with a mean age of 27 years, which allowed an unbiased, comparative evaluation of clinical parameters, which might be interfered with by the physiological factors that are influenced by increasing age. A study has earlier reported that pregnancy rate in ART is significantly associated with BMI and shows a decline at the BMI cut off of 22–24 kg/m^2^ (25 to 35 year olds) and 18–20 kg/m^2^ (over 35 year olds) in PCOS patients [41]. Since the patients in our studied population belong to the former age group that should have an optimal BMI < 24 kg/m^2^, their BMI of 27.79 kg/m^2^ reflects the risk of poor pregnancy outcomes in ART. Women with PCOS are more likely to suffer from type 2 diabetes in later stages of life due to altered endocrinology and insulin resistance [42]. A 24 year follow-up study has shown that 19% of PCOS patients developed type 2 diabetes in comparison to 1% of controls during the time course [43]. Our results agree with the observations that link PCOS as a significant risk to type 2 diabetes as considerably high fasting glucose levels and insulin resistance were seen in PCOS patient samples. A notable androgen excess with high testosterones was also found in PCOS patients along with elevated progesterone and FSH levels, though no significant difference in estradiol levels were observed between patients and controls. As previously reported, hyperandrogenism is a hallmark of PCOS disease and studies also link such an abnormal steroidogenesis with genetic and environmental factors [44]. Estrogen hormone binds to estrogen receptors ERα and ERβ for its action on genomic expression. Certain estrogen receptor gene variants have been previously linked to the susceptibility of PCOS disease, including estrogen receptor beta gene +1730 G/A polymorphism [45]. As mentioned earlier, studies have shown that in ESR1, the most investigated polymorphisms include rs2234693 (T/C), that is identified by the restriction site of PvuII, and rs9340799 (A/G), which is determined by the restriction site of XbaI in intron 1, and a (TA)n dinucleotide repeat polymorphism in the regulatory regions. A study on the clinical significance of these polymorphisms demonstrated that when two to three consecutive rounds of IVF were evaluated, the PvuII TT genotype was associated with lower pregnancy rates [10,11]. Other studies that observed single cycles found no effect of ESR genotypes on pregnancy rates [13,14,15]. It was also observed that in comparison to PvuII TT patients, PvuII CC patients had better follicular quality, more mature oocytes, a higher fertilization rate, and healthier embryos in ART [11,13,14]. In addition, longer (TA)n repetitions and the PvuII CC genotype together have a better COH response [13]. Whereas, another study on the PvuII and XbaI polymorphisms of ERα found no differences in the distribution of these polymorphisms in patients and healthy subjects; although these variants were associated with insulin resistance and FSH levels, which are indicative of their role as a genetic modifier of the disease [46]. A recent meta-analysis of studies involving 1522 PCOS patients and 4198 controls demonstrated no significant associations between the estrogen receptor gene variants including ESR1 rs2234693, ESR1 rs9340799 and ESR2 rs4936938 polymorphisms [47]. Our study on ESR1 PvuII-rs2234693 polymorphisms also did not show any significant association with the risk and susceptibility to PCOS disease. The differences in serum estradiol levels were also reported to be non-significant. Since there is evidence of estrogen negative feedback on gonadotropin secretion in normal cyclic reproductive endocrinology [48]; and the observations that FSH levels were found to be significantly elevated in PCOS patients might reflect its role as modifier of the disease. In recent years, microRNAs have emerged as the principal regulators of gene expressions through epigenetic phenomena and post-transcriptional interactions with mRNAs; and several of these have been linked to a diseased state [49,50]. As evident from a number of studies, the miRNA-146a rs2910164 loci is a site of a functional polymorphism, which has been linked to the risk of several diseases in different models of inheritance. A study using multiple logistic regression analysis demonstrated an increased risk of esophagogastric junction adenocarcinoma in both males and females with miRNA-146a rs2910164 C>G polymorphism, and no history of smoking and alcohol consumption [51]. A meta-analysis that involved 59,098 subjects showed a correlation of the rs2910164 locus in miR- 146a with the risk of digestive-system cancer in a dominant model [52]. Another study has recently reported that the polymorphism of miRNA-146a rs2910164GC is associated with an increased risk of acquiring infections, through modulation of Notch-1/IL-6 signaling in immune responses [53]. Similarly, a positive association has been reported for the C allele of the miRNA-146a rs2910164 as a predisposing factor for the risk of and susceptibility to the metabolic syndrome [54]. miR- 146a is also associated with inflammation, insulin resistance and its elevated levels have been demonstrated in the follicle fluid of PCOS patients [55]. Our study reports an increased risk of polycystic ovary syndrome associated with CG, GG genotypes and the G allele of the miR- 146a gene variation. An earlier study has also implicated the miR- 146a rs2910164 gene variation in the risk and susceptibility to PCOS [56]. Although the study also shows the CG genotype to be of significance in the associated risk, it was the C allele, which was reported to increase the risk of PCOS. Nevertheless, both the studies demonstrate the miR- 146a rs2910164 gene variation as a functional polymorphism associated with the risk of PCOS. The differences in the allelic forms might be attributed to the ethnicity of the studied population, as it is well established that population-based allele frequencies of polymorphisms that are associated with the risk of certain disease may vary in different ethnic groups [57]. The limitations of the study may include small sample size, which might be inadequate for assessing statistical interaction when the polymorphism is treated as an effect modifier. Furthermore, we did not study the expression of the selected gene. However, the study is important in relating the association of certain gene variations with the risk and susceptibility to PCOS and might contribute to a better understanding of the disease in terms of biomarkers, pathophysiology and personalized medicine in the concerned population.

## Figures and Tables

**Table 1:** ARMS primers for the genotyping of estrogen receptor-1 (ESR1 PvuII-rs2234693 T>C) and miR- 146a-rs2910164 C>G.

Direction		Sequence of Primer	PCR Product	Annealing Temperature
**ESR1 PvuII-rs2234693 T>C ARMS primers**				
ESR1-Fo	FO	5′-TGATATCCAGGGTTATGTGGCAA-3′	278 bp	58 °C
ESR1-Ro	RO	5′-CTGCACCAGAATATGTTACCTATAAAAA-3′		
ESR1-FI-C	FI	5′-TGAGTTCCAAATGTCCCAGCC-3′	193 bp	
ESR1-RI-T	RI	5′-GGGAAACAGAGACAAAGCATAAACA-3′	131 bp	
**miR- 146a-rs2910164 C>G ARMS primers**				
miR- 146a Fo	FO	5′-GGCCTGGTCTCCTCCAGATGTTTAT-3′	364 bp	61 °C
miR- 146a Ro	RO	5′-ATACCTTCAGAGCCTGAGACTCTGCC-3′		
miR- 146a FI-C	FI	5′-ATGGGTTGTGTCAGTGTCAGACCTC-3′	169 bp	
miR- 146a RI-G	RI	5′-GATATCCCAGCTGAAGAACTGAATTTCAC-3′	249 bp	

**Table 2:** Biochemical comparison of study subjects: patients and controls.

Characteristic	Controls ^a^	Cases ^a^	*p* ^b^
***Age and BMI***			
Age ^c^	27.49 ± 4.29	27.89 ± 4.97	0.229
BMI (kg/m2) ^c^	25.71 ± 2.39	27.79 ± 4.82	<0.001
***T2DM Markers***			
Free Insulin (mU/mL) ^c^	8.30 ± 2.79	14.47 ± 6.48	<0.001
HOMA-IR ^c^	1.64 ± 0.68	5.24 ± 3.24	<0.001
FBG (mmol/l) ^c^	5.69 ± 0.93	7.66 ± 2.34	<0.001
***Lipid Markers***			
Triglycerides (mmol/l) ^c^	1.82 ± 0.63	3.58 ± 1.39	0.038
Cholesterol (mmol/l) ^c^	1.36 ± 0.28	1.57 ± 0.37	<0.001
LDL (mmol/l) ^c^	3.88 ± 0.48	5.51 ± 1.47	<0.001
HDL (mmol/l) ^c^	1.55 ± 0.57	1.70 ± 0.85	<0.001
***Endocrine Markers***			
LH (mIU/mL) ^d^	0.08 (0.07–1.38)	3.88 (0.78–9.18)	<0.001
Progesterone (ng/mL) ^d^	17.36 (2.58–19.87)	19.87 (1.77–34.87)	<0.001
FSH (mIU/mL) ^d^	0.41 (0.36–3.56)	5.47 (2.20–6.80)	<0.001
Estradiol (pmol/l) ^d^	238.90 (141.88–488.18)	251.40 (172.97–509.14)	0.167
Testosterone (ng/dl) ^d^	13.98 (8.50–39.58)	62.17 (44.89–92.36)	<0.001

^a^ 102 PCOS cases and 115 healthy controls; ^b^ Student’s *t*-test for continuous variables, Mann–Whitney U-test for variables that were not normally distributed; ^c^ Values as mean ± SD.; ^d^ Values presented as median (interquartile range).

**Table 3:** Association of ESR1 PvuII-rs2234693 C>T between PCOS cases and controls.

Subjects	N = 217	CC	CT	TT	C Allele	T Allele	Df	X^2^	*p* Value
PCOS Cases	102	10(9.80%)	52(50.98%)	40(39.21%)	0.36	0.64	2	1.18	0.55
Controls	115	13(11.30%)	65(56.52%)	37(32.17%)	0.56	0.44			

**Table 4:** Multivariate analysis to study correlation between ESR1 PvuII-rs2234693 C>T gene variability and PCOS risk.

Genotypes	Healthy Controls	PCOS Patients	OR (95% CI)	Risk Ratio (RR)	*p*-Value	
	(N = 115)	(N = 102)				
Codominant						
ER-CC	13	10	(ref.)	(ref.)		
ER-CT	65	52	1.04 (0.42–2.56)	1.01 (0.68–1.50)	0.93	NS
ER-TT	37	40	1.40 (0.55–3.59)	1.17 (0.76–1.80)	0.47	NS
Dominant						
ER-CC	13	10	(ref.)	(ref.)		
ER-(CT + TT)	102	92	1.17 (0.49–2.802)	1.07 (0.73–1.57)	0.071	NS
Recessive						
ER-(CC + CT)	78	62	(ref.)	(ref.)		
ER-TT	37	40	1.36 (0.77–2.375)	1.15 (0.88–1.52)	0.27	NS
Allele						
ER-C	91	72	(ref.)	(ref.)		
ER-A	**139**	**132**	1.36 (0.77–2.37)	1.01 (0.90–1.30)	0.35	NS

**Table 5:** Association of miR- 146a rs2910164 C>G gene variation in PCOS cases and controls.

Subjects	N = 207	GG	GC	CC	Df	X^2^	G	C	*p* Value
Cases	100	27(27%)	43(43%)	30(30%)	2	9.25	0.48	0.52	*0.009*
Controls	107	49(45.79%)	40(37.38%)	18(16.82%)			0.64	0.36	

**Table 6:** Risk association of miR- 146a rs2910164 C>G genotypes with PCOS cases and controls utilizing multivariate analysis.

Genotypes	Healthy Controls	PCOS Cases	OR (95% CI)	Risk Ratio (RR)	*p*-Value
	(N = 107)	(N = 100)			
Codominant					
miR- 146-CC	49	27	(ref.)	(ref.)	
miR- 146-CG	40	43	1.95 (1.03–3.68)	1.33 (1.01–1.76)	0.039
miR- 146-GG	18	30	3.02 (1.42–6.40)	1.71 (1.15–2.56)	0.003
Dominant					
MiR- 146-CC	49	27	(ref.)	(ref.)	
MiR- 146-(CG + GG)	58	73	2.28 (1.27–4.09)	1.45 (1.12–1.87)	0.003
Recessive					
MiR- 146-(CC + GC)	89	70	(ref.)	(ref.)	
MiR- 146-GG	18	30	2.11 (1.09–4.11)	1.49 (1.01–2.20)	0.026
Allele					
miR- 146-C	138	97	(ref.)	(ref.)	
miR- 146-G	76	103	1.92 (1.30–2.85)	1.38 (1.13–1.69)	0.001

## Data Availability

All the data associated with the current study has been presented in this manuscript.
